# Inter-rater agreement for the annotation of neurologic signs and symptoms in electronic health records

**DOI:** 10.3389/fdgth.2023.1075771

**Published:** 2023-06-13

**Authors:** Chelsea Oommen, Quentin Howlett-Prieto, Michael D. Carrithers, Daniel B. Hier

**Affiliations:** ^^1^^ Department of Neurology and Rehabilitation, University of Illinois at Chicago, Chicago, IL, United States; ^^2^^ Department of Electrical and Computer Engineering, Missouri University of Science and Technology, Rolla, MO, United States

**Keywords:** natural language processing, annotation, electronic health records, phenotype, clinical concept extraction, inter-rater agreement, neural networks, signs and symptoms

## Abstract

The extraction of patient signs and symptoms recorded as free text in electronic health records is critical for precision medicine. Once extracted, signs and symptoms can be made computable by mapping to signs and symptoms in an ontology. Extracting signs and symptoms from free text is tedious and time-consuming. Prior studies have suggested that inter-rater agreement for clinical concept extraction is low. We have examined inter-rater agreement for annotating neurologic concepts in clinical notes from electronic health records. After training on the annotation process, the annotation tool, and the supporting neuro-ontology, three raters annotated 15 clinical notes in three rounds. Inter-rater agreement between the three annotators was high for text span and category label. A machine annotator based on a convolutional neural network had a high level of agreement with the human annotators but one that was lower than human inter-rater agreement. We conclude that high levels of agreement between human annotators are possible with appropriate training and annotation tools. Furthermore, more training examples combined with improvements in neural networks and natural language processing should make machine annotators capable of high throughput automated clinical concept extraction with high levels of agreement with human annotators.

## Introduction

Extracting medical concepts from electronic health records is key to precision medicine (1). The signs and symptoms of patients (part of the patient phenotype) are generally recorded as free text in progress notes, admission notes, and discharge summaries (2). Clinical phenotyping of patients involves the mapping of free text to defined terms that are concepts in an ontology (3,4). This is a two-step process that involves identifying appropriate text spans in narratives and then converting the text spans to target concepts in an ontology (5,6). The process of mapping free text to defined classes in an ontology, illustrated in (1) and (2), has been termed **normalization** (7,8).

(1) patientmovementswereataxic⇒ataxia⇒UMLSCUI:C0004134

(2) freetext⇒clinicalconcept⇒machinereadablecode

In this example 1, an annotator highlights the term ataxic, then it is mapped to the concept ataxia, and the UMLS code CUI C0004134 is retrieved (9). This is a slow and error-prone process for human annotators. Agreement between human raters for annotation of clinical text is often low. A study on the agreement for SNOMED CT codes between coders from three professional coding companies yielded about 50 percent agreement for exact matches with slightly higher agreement when adjusted for near matches (10). Another study of SNOMED CT coding of ophthalmology notes yielded low levels of inter-rater agreement ranging from 33 to 64% (11). Identified sources of disagreement between coders included human errors (lack of applicable medical knowledge, lack of recognition of abbreviations for concepts, and general carelessness), annotation guideline flaws (under specified and unclear guidelines), ontology flaws (polysemy of coded concepts), interface term issues (inconsistent categorization of clinical jargon), and language issues (interpretation difficulties due to use of ellipsis, anaphora, paraphrasing, and other linguistic concepts) (12).

The goal of high throughput phenotyping is to use natural language processing (NLP) to automate the annotation process (13). Approaches to high throughput clinical concept extraction have included rule-based systems, traditional machine learning algorithms, deep learning algorithms, and hybrid methods that combine algorithms (6). Tools for concept extraction based on rules, linguistic analysis, and statistical models, such as cTAKES and MetaMap, generally have accuracy and recall between 0.38 and 0.66 (5,14,15). Neural networks are being used for concept recognition with increasing success. Arbabi et al. developed a convolutional neural network that matches input phrases to concepts in the Human Phenotype Ontology with high accuracy (16). Other deep learning approaches, including neural networks based on bidirectional encoder representations from transformers (BERT), show promise for automated clinical concept extraction (5,6,17,18).

In this paper, we examine inter-rater agreement for text-span identification of neurological concepts in notes from electronic health records. In addition to the agreement between human annotators, we examine the agreement between human annotators and a machine annotator based on a convolutional neural network.

## Methods

### Annotation tool

**Figure 1:**
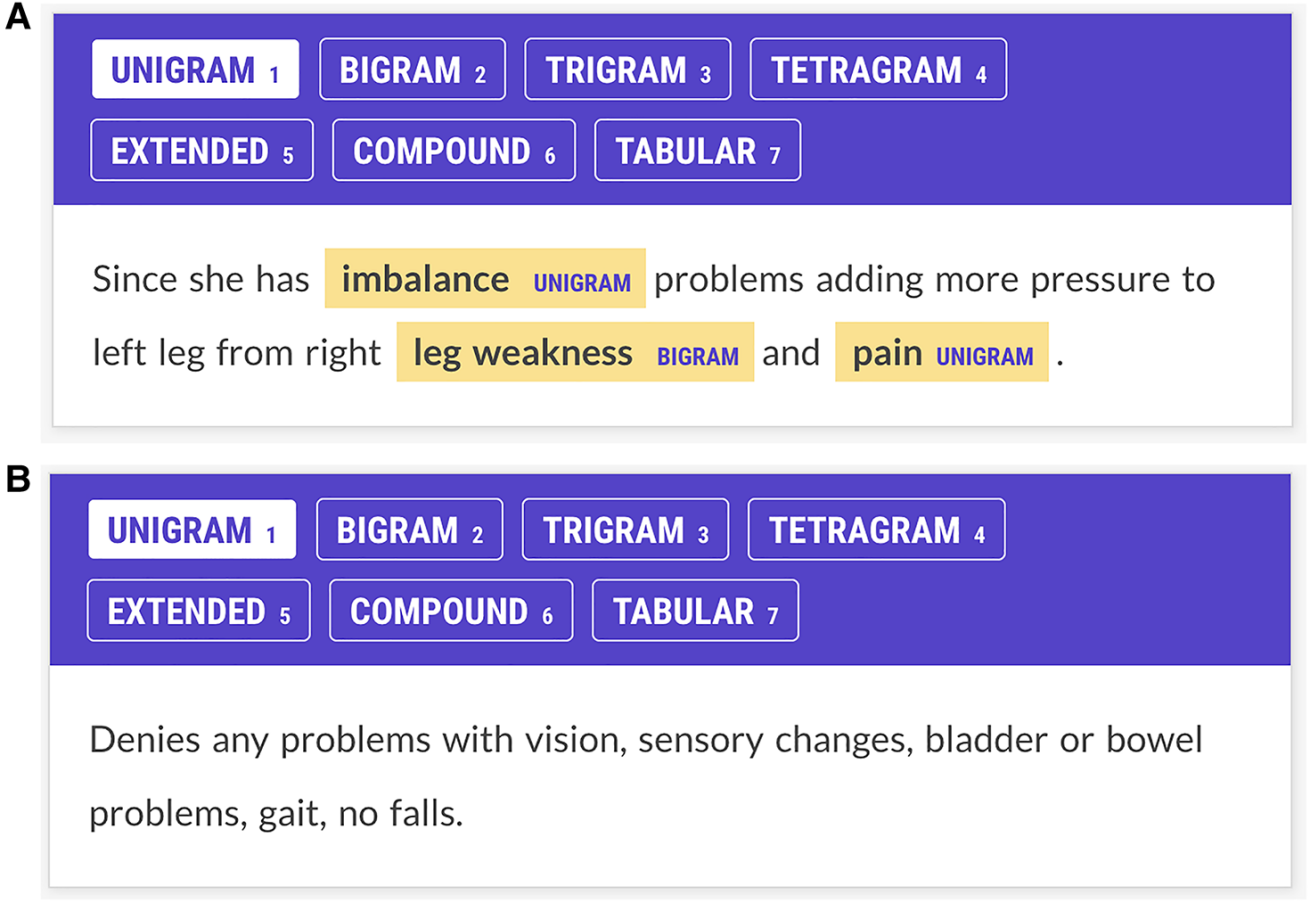
(**A**) Annotator screen for a patient with multiple sclerosis. The patient complains of imbalance, leg weakness, and pain, and these concepts have been annotated. Imbalance and pain are labeled as unigrams; leg weakness is labeled a bigram. Annotators were trained to ignore laterality (e.g., right leg weakness.) Each Prodigy screen reflects one line of text from the JSON input file. This screen has three potential items to contribute to the Kappa statistic: imbalance, leg weakness, and pain. (**B**) Annotator screen for neurological concepts for a patient with multiple sclerosis. The patient denies problems with vision, sensation, bladder, bowel, gait, or falls. The annotators are trained not to annotate negated concepts. The NN had no specific negation rule but learned not to tag negated concepts through training examples. Since there are no signs and symptoms in this screen, if both annotators show no annotations, a score of 1 is assigned to the Kappa statistic for agreement on this screen. If one annotator shows no annotations and another shows annotations on this screen, annotator disagreement is scored.

Prodigy (Explosion AI, Berlin, Germany) was used to annotate neurologic concepts in the EHR physician notes. Prodigy runs under python in the terminal mode of macOS, Windows, or Linux. It creates a web interface locally (Figures 1A,B). As input, Prodigy requires free text to be converted to JSON format.

(3) $$\left\{\;\;\;{}^{\prime }{}^{\prime }\;\;{\mathrm{text}}^{\prime \prime }\;:\;\;\;\;{}^{\prime }{}^{\prime }\;\;\mathrm{The}\;\mathrm{patient}\;\mathrm{had}\;weakness\;\mathrm{and}\;{sensory\;loss}^{\prime \prime }\right\}$$

Each line of text from a JSON file 3, appears as a separate screen for annotation by Prodigy (Figures 1A,B). Annotations are stored in an SQLite database and are exportable with annotations and text spans as a JSON file. Prodigy is integrated with the *spaCy* natural language processing toolkit (Explosion AI) and can train neural networks for named entity recognition and text classification.

The Kappa statistic was used to assess agreement between the three annotators and the neural network. The Kappa statistic corrects observed rater agreement for chance rater agreement. It ranges from 0 to 1, where 1 is complete agreement, 0 is a chance agreement. Values of Kappa of 0.6 to 0.79 are considered substantial agreement, values between 0.8 and 0.90 are considered strong agreement, and values over 0.90 are considered near perfect agreement (19,20). For each line of text that had one or more annotations (3), the agreement was rated 1 for the annotations if both annotators agreed and rated 0 if the annotators disagreed. A line of text with no annotations (null_annotations) by either annotator was scored 1 for agreement. The total number of annotations considered by the Kappa statistic for two raters **A** and **B** was (A∪B+null_annotations).

### Rater training and instructions

Three annotators participated in the research. Annotator 1 (A1) was a senior neurologist, Annotator 2 (A2) was a pre-medical student majoring in neuroscience, and Annotator 3 (A3) was a third-year medical student. Raters first reviewed neurologic signs and symptoms in the neuro-ontology of neurological concepts (21) and then were instructed to find all neurological concepts in the neurology notes. Signs and symptoms (ataxia, fatigue, weakness, memory loss, etc.) were annotated but not disease entities (Alzheimer’s disease, multiple sclerosis, etc.) Raters annotated the neurologic concepts and ignored laterality and other modifiers (e.g., *arm pain* for *right arm pain*, *back pain* for *severe back pain*, etc.) In addition, annotators tagged each text span with an category label (see Figures 1A,B). Category labels included *unigrams* (one-word concepts such as ataxia), *bigrams* (two-word concepts such as double vision), *trigrams* (three-word concepts such as low back pain), *tetragrams* (four-word concepts such as relative afferent pupil defect), *extended* (text span annotations longer than four words), *compound* (multiple concepts in one text span such as brisk ankle and knee reflex), and *tabular* (concepts represented in tabular or columnar format, usually showed right and left body sides). Our motivation for tagging signs and symptoms by the length and type of the text span was a hypothesis that neural networks trained to recognize signs and symptoms in medical text would exhibit lower accuracies with longer text spans. This hypothesis was confirmed by a recent study from our group (18).

### The machine annotator

The machine annotator (NN) was a neural network that was trained to recognize text spans containing neurology concepts in the electronic health record physician notes. The NN was the default spaCy named entity recognition model based on a four-layer convolutional neural network (CNN) that looked at four words on either side of each token using *tok2vec* with an initial learning rate $1\times 10^{-3}$. The default parameters provided by Prodigy were used for training. NN was trained on 11,000 manually annotated sentences derived from neurology textbooks, online neurological disease descriptions, and electronic health record notes. Further details on training the NN are available in (18).

### Annotations

Five patient EHR notes were annotated for each of the three rounds. The annotation of EHR clinical notes for research purposes was approved by the Institutional Review Board of the University of Illinois (UIC Neuroimmunology Biobank 2017-0520Z). Informed patient consent for use of clinical notes was obtained from all subjects through the UIC Biobank Project. Three human annotators (A1, A2, and A3) and the machine annotator (NN) annotated each note. After each round, the annotators met and reviewed any annotation disagreements. The annotations of each annotator were stored in an SQLite database and exported as a JSON file for scoring for inter-rater agreement in Python. Text spans were mapped to concepts in the neuro-ontology (21) utilizing a lookup table with 3,500 target phrases and the similarity method from spaCy (22) (pp. 152–54). Univariate analysis of variance and Cohen’s Kappa statistic were calculated with SPSS (IBM, version 28).

## Results

Annotators identified neurological signs and symptoms in physician notes from electronic health records. Each annotator identified the text span associated with each sign and symptom and assigned a category label to each annotation (e.g., unigram, bigram, trigram, etc.) Inter-rater agreement (adjusted and unadjusted) was calculated between the three human annotators and the machine annotator (NN).

Although five EHR notes were annotated for each round, the notes varied in length. Each line in the EHR note was converted to a single line in the JSON file and generated one annotation screen in the Prodigy annotator. Round 1 had 625 annotation screens with 139 signs and symptoms to annotate, Round 2 had 674 annotation screens with 205 signs and symptoms to annotate, and Round 3 had 523 annotation screens with 138 signs and symptoms to annotate. Since the number of signs and symptoms was less than the number of annotation screens, many annotation screens had no signs or symptoms to annotate (null screens). When both annotators agreed that the annotation screen had no signs or symptoms, this was scored as annotator agreement for both the adjusted and unadjusted metrics (Kappa and concordance).

Concordance (unadjusted agreement) on the text span task was 88.9%±3.2 (mean±SD) between the human annotators and was 83.9%±4.6 (mean±SD) between the human annotators and the machine annotator (human-human mean was higher, one-way ANOVA, df=1, p=0.016). Concordance (unadjusted agreement) on the category label task was 87.7%±4.4 (mean±SD) between human annotators and was 84.6%±5.5 (mean±SD) between the human annotators and the machine annotator (means did not differ, one-way ANOVA, df=1, p=0.212).

**Figure 2:**
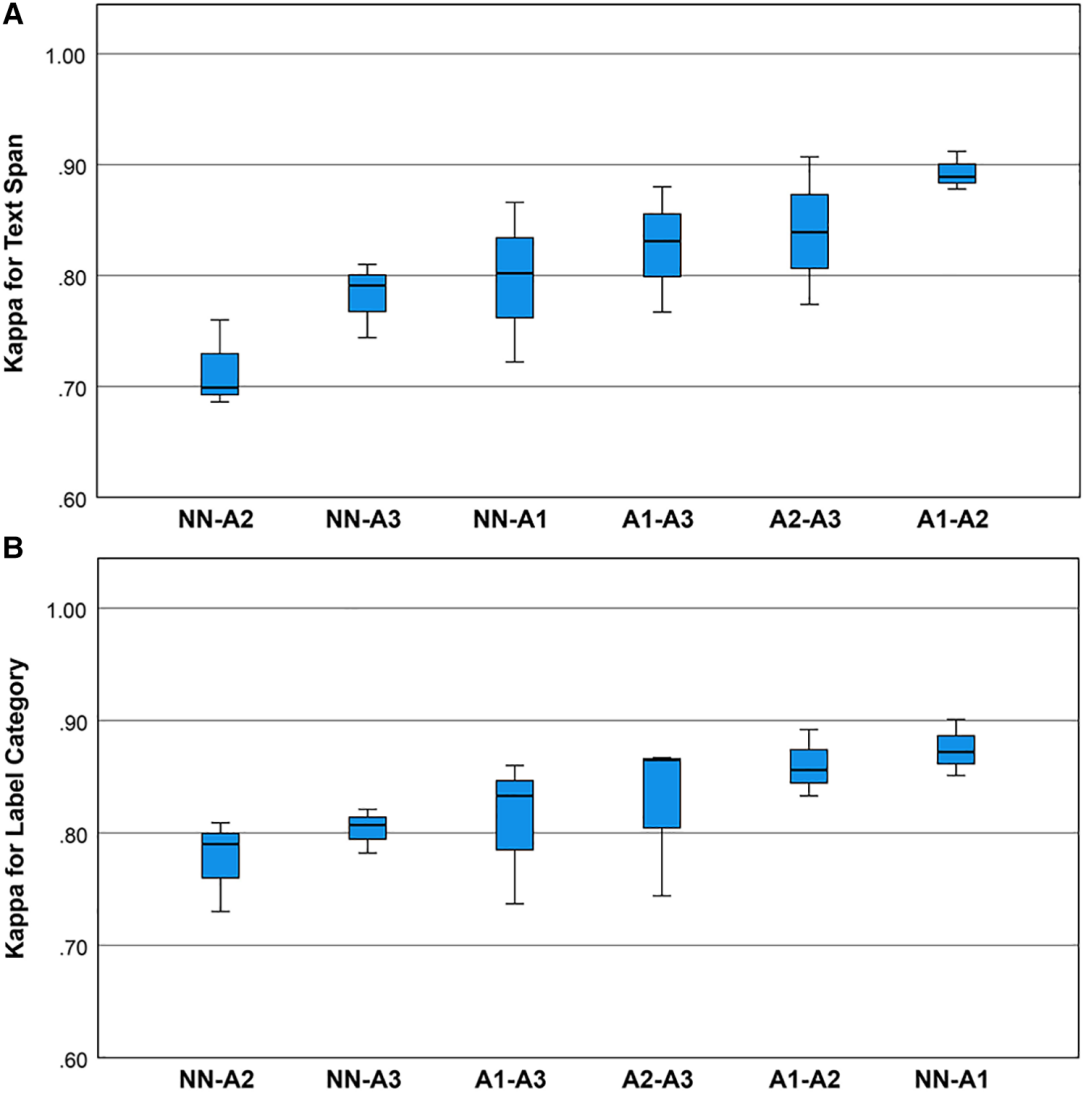
(**A**) Boxplots for the Kappa statistic for inter-rater agreement for text spans for the neurological concepts. Univariate analysis of variance showed that mean inter-rater agreement differed by rating pair (one-way ANOVA, df=5, p=0.021). Post hoc comparisons by the Bonferroni method showed that pair A1-A2 outperformed pair NN-A2. (**B**) Boxplots for the Kappa statistic for inter-rater agreement for category labels for the neurological concepts. Univariate analysis of variance showed that mean Kappa for category label agreement did not differ by rating pair (one-way ANOVA, p=0.165, df=5).

**Figure 3:**
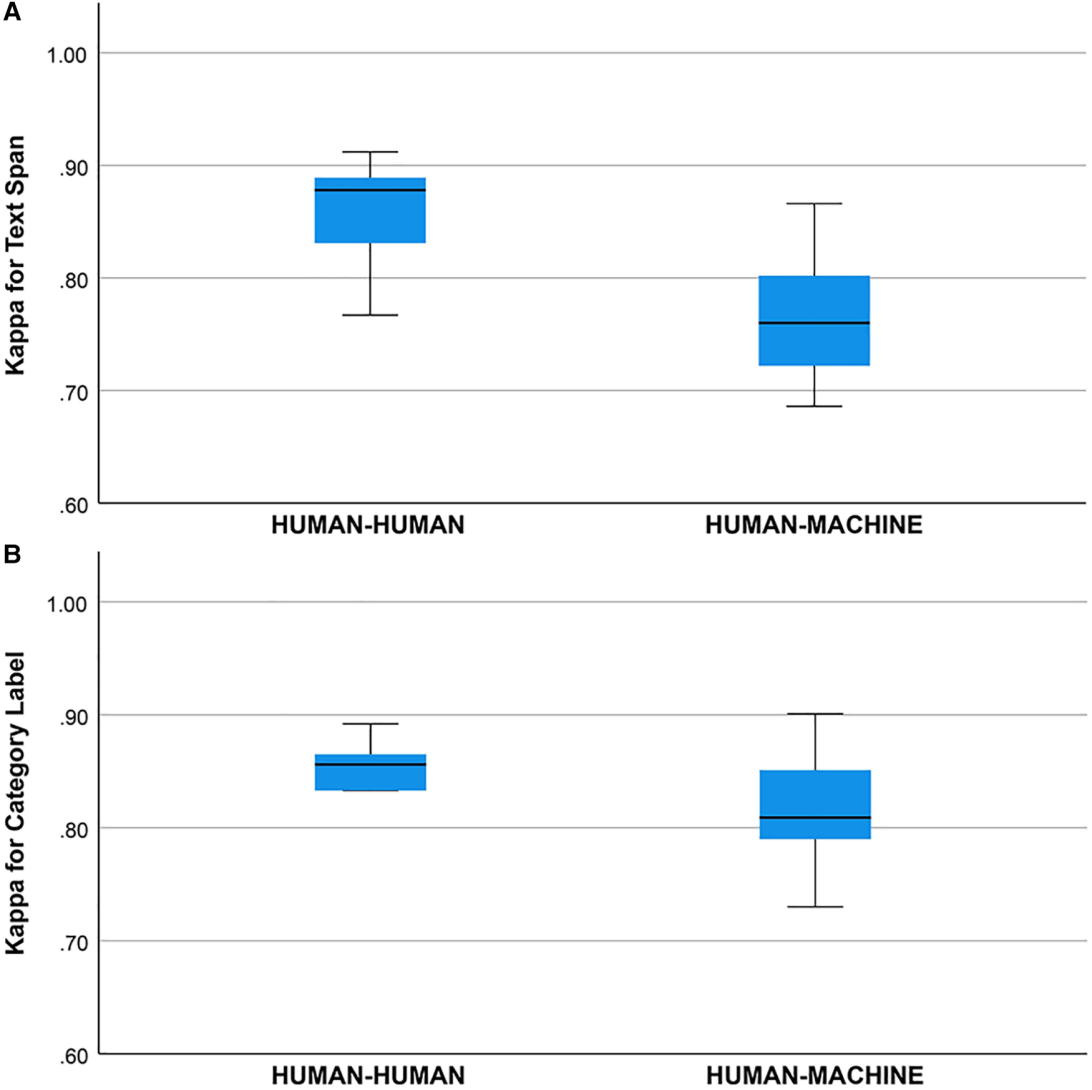
(**A**) Kappa statistic for agreement between human-human and human-machine raters for text span. Groups differed, one-way ANOVA, df=1, p=0.004. (**B**) Kappa statistic for agreement between human-human and human-machine raters for category label. Groups did not differ, one way ANOVA, df=1, p=0.589.

**Figure 4:**
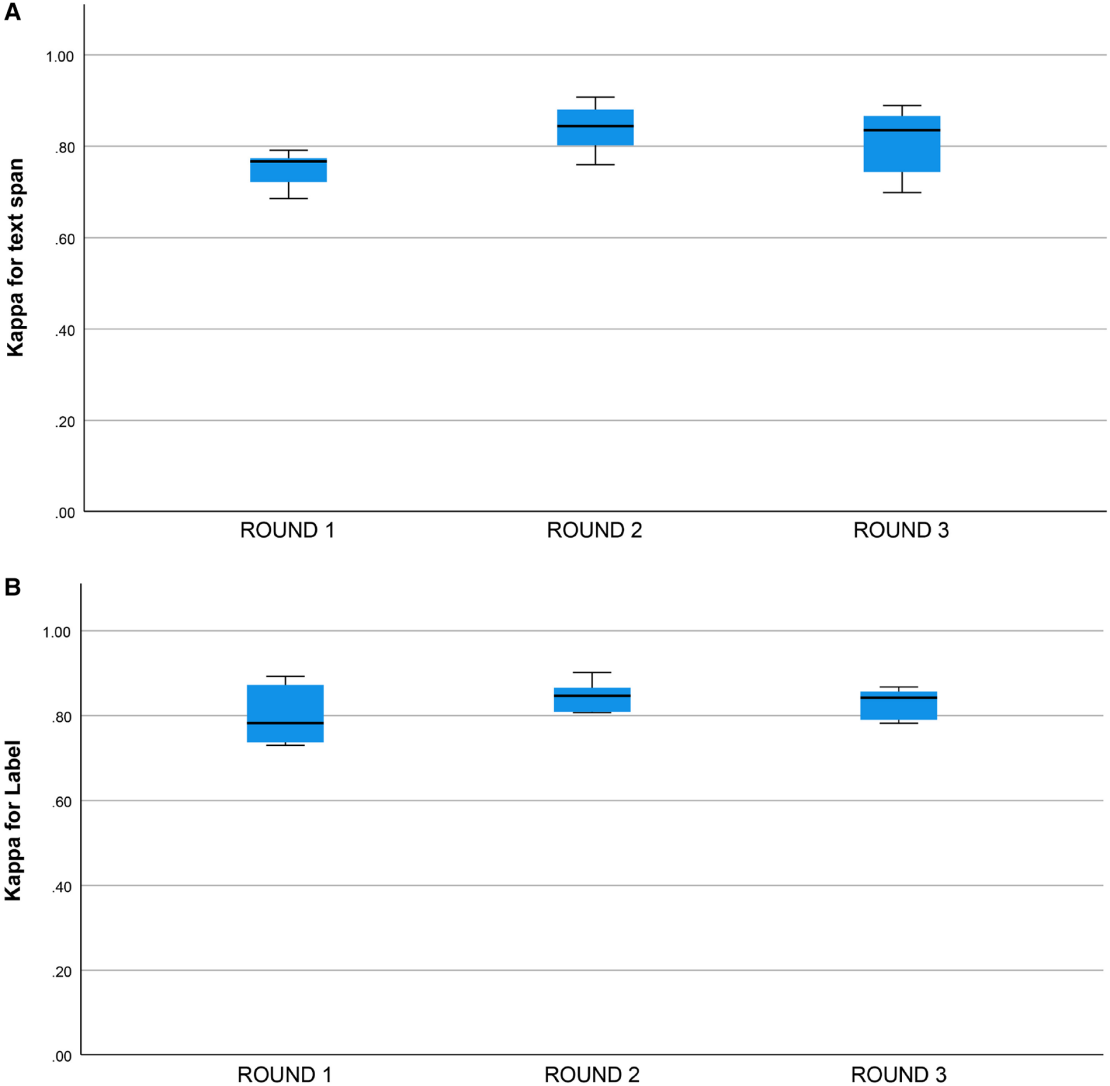
(**A**) Kappa statistic for inter-rater agreement for text span by round. Round 1: 0.78±0.03 (mean±SE), Round 2: 0.84±0.03, Round 3: 0.81±0.03, groups do not differ, one-way ANOVA, df=2, p=0.310. (**B**) Kappa statistic for inter-rater agreement for category label by round. Round 1: 0.80±0.21 (mean±SE). Round 2: 0.85±0.21, Round 3: 0.83±0.21, groups do not differ, one-way ANOVA, df=2, p=0.306.

Cohen’s Kappa statistic (κ) was high for both the text span task (0.715 to 0.893) and the category label task (0.72 to 0.89) (Figures 2A,B). On the text span identification task (Figure 3A) κ was higher for the human-human pairs (0.85±0.05 mean±SD) than the human-machine pairs (0.76±0.06). On the category label task, κ (Figure 3B) was similar between the human-human pairs (0.83±0.05 mean±SD) and the human-machine pairs (0.82±0.06). κ for the text span task and the category label task did not differ by round (for p values and means see Figures 4A,B).

## Discussion

Signs and symptoms are an important component of a patient’s phenotype. Extracting these phenotypic features from electronic health records and converting them to machine-readable codes makes them computable (23). These computable phenotypes are critical to precision medicine initiatives (24–26). Agrawal et al. (5) have conceptualized clinical entity extraction as a two-step process of text span recognition followed by clinical entity normalization. Text span recognition is the identification of signs and symptoms in the free text; entity normalization is the mapping of this text to canonical signs and symptoms in an ontology such as UMLS (9). We have focused on an inter-rater agreement for text span annotation. For entity normalization, we depended on a look-up table that mapped text spans to concepts in neuro-ontology. We found high inter-rater concordance (unadjusted agreement) among the human annotators (approximately 89%) with a lower concordance (unadjusted) agreement between the human annotators and the machine annotator (approximately 84%).

The concordance (unadjusted agreement) for category labels was lower than the inter-rater agreement for text spans which may have been due to factors such as the use of hyphens in the free text of the EHR notes and annotator uncertainty about which types of text spans required the tabular label. The Kappa statistic (adjusted agreement) for human-human raters was between 0.77 and 0.91, and the Kappa statistic for the human-machine agreement was between 0.69 and 0.87 (Figure 3A). We consider the inter-rater adjusted agreement between the human raters (0.77 to 0.91) good, especially when contrasted with the inter-rater adjusted agreement between trained neurologists eliciting patient signs and symptoms (27,28). For trained neurologists eliciting signs and symptoms such as weakness, sensory loss, ataxia, aphasia, dysarthria, and drowsiness, the κ statistic ranges from 0.40 to 0.70 (27,28).

The higher levels of agreement in this study may reflect that eliciting a sign or symptom from a patient is more difficult than annotating a sign or symptom in an EHR. Nonetheless, the adjusted agreement (κ) was higher in this study than in prior annotation studies (10,11), possibly reflecting the training of the annotators, the use of a neuro-ontology, the decision not to code severity or laterality of the symptoms, and the use of a sophisticated annotation tool.

We did not find a training effect for the human annotators across rounds (Figures 4A,B). Although the annotators met after each round and discussed discrepancies in their annotations, inter-rater adjusted and unadjusted agreement did not improve significantly between rounds. This suggests that there may be a ceiling for inter-rater agreement for text span annotation with a Kappa of 0.80 to 0.90 and that higher levels of agreement may not be possible due to the complexity of the task and random factors that are not addressable with additional training or experience. This ceiling effect for the human inter-rater agreement has implications for the potential for higher rates of inter-rater agreement between humans and machines (Figure 3B). Mean inter-rater adjusted agreement for text span was higher for the human-human pairs (κ=0.85) than the human-machine pairs (κ=0.76). Additional training examples would likely improve the performance of the machine annotator on the text span and category label tasks. Furthermore, other neural networks are likely to outperform the convolutional neural network (CNN), which is the baseline for Prodigy. We have found that a neural network based on bidirectional encoder representations from transformers (BERT) can improve performance on the text span task by 5 to 10% (18). Others have found that deep learning approaches based on BERT outperform approaches based on CNN for concept identification and extraction tasks (17). A ceiling effect for inter-rater agreement for annotating signs and symptoms, whether human-human or human-machine, near a κ of 0.90 is likely.

Given the heavy documentation burden on physicians and physician burn-out attributed to electronic health records, physician documentation of signs and symptoms will likely continue as free text. Structured documentation of signs and symptoms as an alternative to free text is too burdensome in the current environment (29–34). A medium-sized medical center with a daily inpatient census of 300 and a daily outpatient census of 2,000 generates at least 5,000 clinical notes daily or over 1.5 million notes annually (unpublished estimates based on two academic medical centers). The sheer volume of clinical notes in electronic health records makes the manual annotation of signs and symptoms impractical. Extracting signs and symptoms for precision medicine initiatives will depend on advances in natural language processing and natural language understanding.

Although high throughput phenotyping of electronic health records by manual methods is impractical (13), the manual annotation of free text in electronic health records can be used to train neural networks for phenotyping. Neural networks can also speed up the manual annotation process. The annotator Prodigy (35,36) has an annotation mode called *ner.correct*, which uses a trained neural network to accelerate the manual annotation of signs and symptoms.

With suitable training and guidelines, high levels of inter-rater agreement between human annotators for signs and symptoms are feasible. Restricting the annotation to a limited domain (e.g., neurological signs and symptoms) and restricted ontology (e.g., neuro-ontology) simplifies manual annotation. Although the inter-rater agreement between human and machine annotators was lower than between human annotators, advances in natural language processing should bring inter-rater agreement between machines and humans closer and make high throughput phenotyping of electronic health records feasible.

This work has limitations. The sample of clinical notes was small (five patient notes per annotation round). A larger sample of notes would have been desirable. The annotation process was restricted to neurological signs and symptoms in neurology notes. The target ontology was a limited neuro-ontology with 1600 concepts (21). We evaluated only one machine annotator based on a convolutional neural network. Other neural networks are likely to perform better. Our results on an inter-rater agreement might not generalize to other medical domains and ontologies. Although we had three raters for this study, we did not designate any of them as the “gold standard,” and we elected to calculate inter-rater agreement for each pair of raters separately. In our opinion, unadjusted agreement at the 90% level between human raters should be considered high. Likewise, machine annotators that can reach 90% unadjusted agreement with human annotators should be considered accurate. Because we lacked a gold standard, we chose to measure the performance of the machine annotator as concordance (unadjusted agreement) and Kappa statistic (adjusted agreement) rather than as accuracy, precision, and recall. Although we used ANOVA to assess the significance of differences in the means for adjusted and unadjusted agreement, we cannot be certain that all assumptions underlying ANOVA were met in our samples, including normality, homogeneity of variance, and independence.

## Data Availability

The raw data supporting the conclusions of this article will be made available by the authors, without undue reservation.
